# Glyceraldehyde caused Alzheimer’s disease-like alterations in diagnostic marker levels in SH-SY5Y human neuroblastoma cells

**DOI:** 10.1038/srep13313

**Published:** 2015-08-25

**Authors:** Yoshiki Koriyama, Ayako Furukawa, Michiru Muramatsu, Jun-ichi Takino, Masayoshi Takeuchi

**Affiliations:** 1 Graduate School and Faculty of Pharmaceutical Sciences, Suzuka University of Medical Science, Suzuka, Mie, 513-8670, Japan; 2 Department of Pathophysiological Science, Faculty of Pharmaceutical Science, Hokuriku University, Kanazawa, Ishikawa, 920-1181, Japan; 3 Laboratory of Biochemistry, Faculty of Pharmaceutical Sciences, Hiroshima International University, Kure, Hiroshima, 737-0112, Japan; 4 Department of Advanced Medicine, Medical Research Institute, Kanazawa Medical University, Uchinada-machi, Ishikawa, 920-0293, Japan

## Abstract

Clinical evidence has implicated diabetes mellitus as one of the risk factors for the development and progression of Alzheimer’s disease (AD). However, the neurotoxic pathway activated due to abnormalities in glucose metabolism has not yet been identified in AD. In order to investigate the relationship between impaired cerebral glucose metabolism and the pathophysiology of AD, SH-SY5Y human neuroblastoma cells were exposed to glyceraldehyde (GA), an inhibitor of glycolysis. GA induced the production of GA-derived advanced glycation end-products (GA-AGEs) and cell apoptosis, glycolytic inhibition, decreases in the medium concentrations of diagnostic markers of AD, such as amyloid β 1-42 (Aβ42), and increases in tau phosphorylation. These results suggest that the production of GA-AGEs and/or inhibition of glycolysis induce AD-like alterations, and this model may be useful for examining the pathophysiology of AD.

Alzheimer’s disease (AD) is characterized pathologically by the presence of senile plaques (SPs) and neurofibrillary tangles (NFTs), the major constituents of which are the amyloid β protein (Aβ) and tau protein^1^. The accumulation of Aβ is considered to be one of the early and causative events in the pathogenesis of AD and markedly increases during progression of the disease, leading to the generation of NFTs and, ultimately, neuronal cell death^2^. AD is a worldwide health problem with implications for an increasing number of people and countries. Thus, the early diagnosis and treatment of AD is important for delaying the degenerative process and dementia. Reactive derivatives from non-enzymatic sugar-protein condensation reactions, as well as lipids and nucleic acids exposed to reducing sugars including glucose, form a heterogeneous group of irreversible adducts called advanced glycation end-products (AGEs)^3^. The formation and subsequent accumulation of AGEs in various tissues are known to progress during normal aging, and at a markedly faster rate in diabetes mellitus (DM)^4^,^5^. Numerous epidemiological studies have indicated that a relationship exists between DM and AD, with the incidence of AD being up to 2–5-fold higher in patients with DM^6^. A previous study detected AGEs, which were identified immunohistochemically, in SPs and NFTs from patients with AD^7^. Furthermore, we demonstrated that glyceraldehyde (GA)-derived AGEs (GA-AGEs), one of the representative ligands for the receptor of AGEs (RAGE), but not other sugar-derived AGEs, exerted toxic effects on cultured neuronal cells^8^. The neurotoxic effects of DM serum were completely canceled by neutralizing antibodies against GA-AGEs^8^. Therefore, we hypothesized that AGEs, especially GA-AGEs, are one of the missing links between AD and DM.

Cerebrospinal fluid (CSF) tests have been proposed for the early detection of AD^9^. However, there is currently no evidence to suggest that CSF findings in DM patients are an indicator AD. Aβ42 and total and phosphorylated tau protein (p-tauT181) in the CSF are useful markers for the diagnosis of AD, in which Aβ42 concentrations are lower while the tau protein and its phosphorylated form are higher than those in healthy subjects^10^,^11^. A previous study showed that the intracellular phosphorylation of tau was increased in the cortex of AD patients^12^. The CSF levels of vascular endothelial growth factor (VEGF) and transforming growth factor-β (TGF-β) were found to be significantly higher in AD patients than in healthy controls^13^. However, limited information is available for the mechanisms underlying CSF and intracellular molecular changes. Although recent findings suggest that cerebral glucose metabolism is impaired from the early stage of AD^14^,^15^, it currently remains unclear whether reductions in the utilization of glucose lead to the development of AD.

In the present study, SH-SY5Y human neuroblastoma cells were treated with GA, an inhibitor of glycolysis^16^,^17^, and examinations of cell viability and measurements of the concentrations of Aβ42, total tau, and p-tauT181 proteins in culture media as well as the phosphorylation ratio of intracellular tau were then performed in order to determine the involvement of glucose metabolism in the pathophysiology of AD.

## Results

### Toxic effects of GA on SH-SY5Y cells

**Figure 1:**
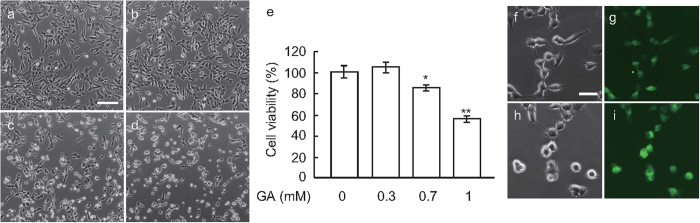
Cytotoxicity of GA in SH-SY5Y cells. (**a**–**d**) Microscopic images of SH-SY5Y cells after a 24 h treatment with GA at 0 (**a**), 0.3 (**b**), 0.7 (**c**), and 1 mM (**d**). Scale bar = 50 μm. (**e**) GA dose-dependently induced cell death in SH-SY5Y cells. *P < 0.05, **P < 0.01 *vs*. 0 mM GA (n = 6). (**f**–**i**) GA-induced apoptosis was observed by staining with green fluorescent YO-PRO®-1 (**g**, 0 mM GA; **i**, 1 mM GA). (**f**) and (**h**) show the same visual field of phase contrast images to (**g**) and (**i**). Scale bar = 20 μm.

A microscopic analysis revealed that GA clearly exerted cytotoxic effects on SH-SY5Y human neuroblastoma cells at 0–1 mM (Fig. 1a–d). The WST-8 assay showed that the viability of cells was significantly reduced from 0.7 mM (Fig. 1e). Fluorescent microscopic analyses revealed that cells exhibited green fluorescence with YO-PRO®-1 dye, which detected early apoptosis in a GA-dose dependent manner (Fig. 1f–i). On the other hand, the red fluorescence of propidium iodide for necrosis was not observed (data not shown), suggesting that GA-induced cell death was mainly caused by apoptosis.

### Characterization of GA-induced cell death in SH-SY5Y cells

**Figure 2:**
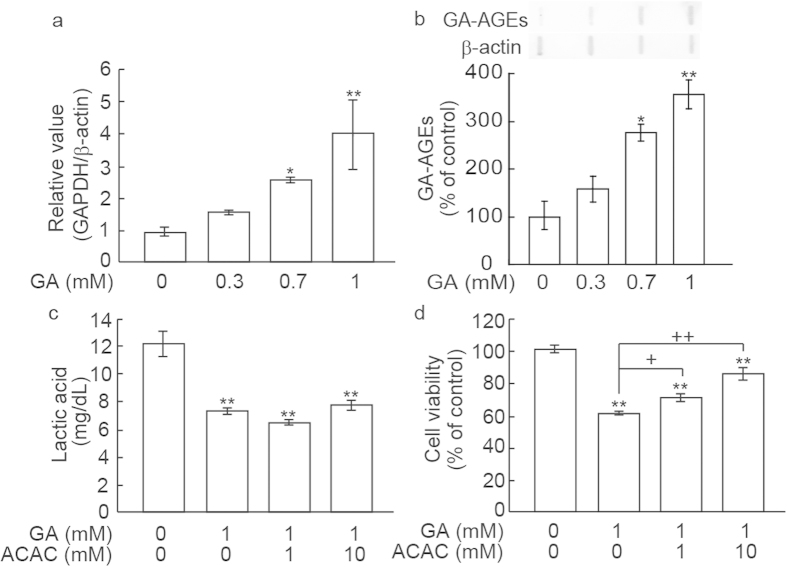
Characterization of GA-induced cytotoxicity in SH-SY5Y cells. (**a**) GAPDH mRNA levels increased in a dose-dependent manner. *P < 0.05, **P < 0.01 *vs*. 0 mM GA (n = 3). (**b**) Production of GA-AGEs by the GA treatment for 24 h. GA-AGEs were measured by slot blotting analyses with an immunopurified anti-GA-AGE antibody. Graphical representation of GA-AGE bands in the slot blot. *P < 0.05, **P < 0.01 *vs*. 0 mM GA (n = 3). (**c**) Evaluation of lactic acid concentrations after the treatment with GA. SH-SY5Y cells were pre-incubated with ACAC at concentrations of 0, 1, and 10 mM for 15 min, then incubated with 1 mM GA for 24 h. **P < 0.01 *vs*. 0 mM GA + 0 mM ACAC (n = 3). (**d**) ACAC prevented GA-induced cytotoxicity. ACAC significantly recovered cell death induced by 1 mM GA in a dose-dependent manner. **P < 0.01 *vs*. 0 mM GA + 0 mM ACAC (n = 6). ^+^P < 0.05, ^++^P < 0.01 *vs*. 1 mM GA + 0 mM ACAC.

A relationship has recently been reported between the expression of glyceraldehyde-3-phosphate dehydrogenase (GAPDH) and apoptotic events^14^. Real-time RT-PCR experiments showed that the expression of GAPDH was significantly increased by the addition of GA (Fig. 2a). These results suggested that GA caused cell toxicity concomitant with increases in the gene expression of GAPDH in SH-SY5Y cells. We then measured GA-AGE levels after the 24-h treatment of SH-SY5Y cells with GA using slot blots. A small band of GA-AGEs was observed in the vehicle control (Fig. 2b). GA dose-dependently increased the production of GA-AGEs in SH-SY5Y cells. The reductions observed in cell viability following a 24 h incubation with GA were significantly restored by the addition of 1 mM (P < 0.05) or 10 mM (P < 0.01) acetoacetate (ACAC), a substrate for the tricarboxylic acid (TCA) cycle (Fig. 2d), whereas lactic acid concentrations were not (Fig. 2c). Glucose consumption, calculated by differences in medium concentrations during the incubation, negatively correlated with lactic acid concentrations (data not shown). These results suggested that GA inhibited glycolysis while ACAC restored cell viability without recovering reductions in glycolysis.

### Effects of GA on Aβ42 and the tau protein and its phosphorylated form in SH-SY5Y cells

**Figure 3:**
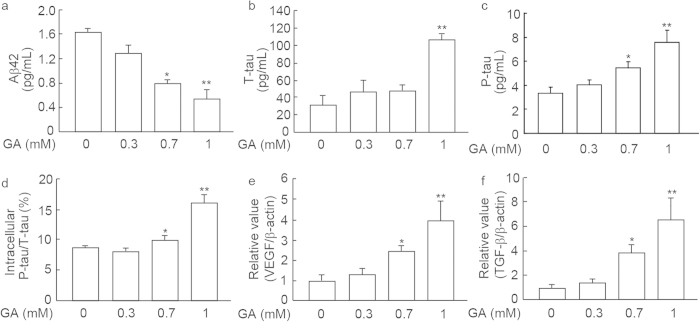
Changes in AD biomarkers after the GA treatment. (**a**-**c**) Changes in AD biomarkers of Aβ42 (**a**), total tau (T-tau, **b**), and p-tauT181 (P-tau, **c**) after a 24 h treatment with GA. *P < 0.05, **P < 0.01 *vs*. 0 mM GA (n = 3). (**d**) Intracellular changes in T-tau and P-tau after a 24 h treatment with GA. GA-dose dependently increased P-tau/T-tau after the GA treatment. *P < 0.05, **P < 0.01 *vs*. 0 mM GA (n = 3). (**e**,**f**) Level changes in other AD biomarkers after the GA treatment. mRNA expression levels were analyzed by real-time PCR after the GA treatment for 24 h. GA significantly increased the levels of VEGF (**e**) and TGF-β (**f**) from a GA concentration of 0.7 mM. *P < 0.05, **P < 0.01 *vs*. 0 mM GA (n = 3).

In order to determine the effects of GA on CSF markers for the development of AD, an aliquot of medium was analyzed by an enzyme-linked immunosorbent assay (ELISA) 24 h after the addition of GA. Aβ42 in the medium decreased in a GA dose-dependent manner (Fig. 3a). In contrast, GA significantly increased tau and its phosphorylated form, p-tauT181 (Fig. 3b,c) in the medium. The ratio of the phosphorylation of intracellular tau was increased significantly at 0.7 mM (P < 0.05) and 1 mM (P < 0.01) GA (Fig. 3d). In addition, VEGF (Fig. 3e) and TGF-β (Fig. 3f), which are also AD biomarkers, were increased when the concentration of GA added was greater than 0.7 mM.

## Discussion

The present study has three salient results: 1) GA induced the formation of GA-AGEs and exhibited cytotoxicity in SH-SY5Y cells. 2) The mechanism underlying GA-induced cell death involved the inhibition of glycolysis and concomitant induction of GAPDH. 3) Changes in the levels of AD biomarkers in GA-treated culture media were consistent with those in the CSF of AD patients.

Epidemiological studies recently reported that the risk of developing AD was higher in DM patients than in the general population^18^. The Rotterdam study, which surveyed more than 6,300 patients, indicated a relationship between DM and AD with a relative risk (RR) of 1.9^19^. Given the recent interest in the relationship between insulin and AD, patients in that study receiving exogenous insulin therapy were at the highest risk (RR 4.3) of developing dementia^20^,^21^. On the other hand, the Honolulu-Asia Aging Study, which explored a cohort of 2,574 patients, also indicated that the risk of developing AD was 1.8 in DM patients while that of vascular dementia was 2.3^22^. Since AGE levels were previously shown to be upregulated in the brains of diabetic patients with AD^23^, these findings may partly explain the clinical link between DM and AD.

The involvement of AGEs in AD has been suggested in several studies published successively between 1994 and 1995^24^,^25^,^26^. SPs were previously shown to be positively stained by an anti-serum against glucose-derived AGEs (Glu-AGEs)^7^. Münch’s group also reported that Glu-AGEs had similar effects to those of Aβ, namely, increases in neurotoxicity and glucose consumption in a SH-SY5Y cell culture system^27^,^28^,^29^. In our previous studies^30^,^31^, we demonstrated that α-hydroxyaldehydes (glyceraldehyde and glycolaldehyde) and dicarbonyl compounds (glyoxal, methylglyoxal, and 3-deoxyglucosone) contributed to the glycation of proteins. We confirmed that GA-AGEs and glycolaldehyde-derived AGEs (Glycol-AGEs) were strongly neurotoxic in a neuronal culture system^1^,^8^. The neurotoxicities of these AGE species were stronger than those of Glu-AGEs and N-(carboxymethyl)lysine (CML), two extensively examined AGE species. Moreover, the neurotoxic effects of serum AGEs from diabetic patients on hemodialysis were completely attenuated by the addition of an anti-GA-AGE-specific antibody, but not the antibodies of glycolaldehyde-, methylglyoxal-, glyoxal-, 3-deoxyglucosone-, or glucose-derived AGEs^1^,^8^. Therefore, GA-AGEs are powerful candidate molecules for neurodegeneration in AD, and, consequently, we focused on the mechanism underlying GA-AGE-induced neurotoxicity in the present study.

On the other hand, a Glu-AGE antibody was shown to react with SPs, mainly with the amyloid core, whereas GA-AGE and Glycol-AGE antibodies showed no immunoreactivity with SPs^32^. These findings suggested that Aβ was glycated by glucose, rather than GA. GA-AGEs are mainly present in the neurons of the hippocampus and parahippocampal gyrus. It was mainly localized in the perikarya of neurons and staining pattern was uniform, differing from the dot-like pattern of Glu-AGE staining^32^. Furthermore, Glu-AGEs were detected in intracellular and extracellular sites, whereas GA-AGEs were only found intracellularly. This discrepancy indicated that the mechanisms underlying the neurotoxicity induced by Glu-AGEs and GA-AGEs differed.

In the present study, we detected GA-AGEs intracellularly in SH-SY5Y cells using slot blots. GA dose-dependently increased GA-AGE levels, as shown in Fig. 2b. We previously investigated which types of AGEs triggered the development of AD pathology using a culture system. We showed that cell viability was markedly decreased by the addition of GA-AGEs^1^,^8^. In the present study, lactic acid concentrations in culture media, to which 1 mM GA had been added, were approximately 50% lower than those in the control and ACAC recovered cell viability from GA-induced cell death. These results suggested that GA induced the inhibition of glycolysis. In a study using positron emission tomography, cerebral glucose metabolism was found to be lower in AD patients than in healthy controls^14^. Needham *et al*. reported that GA attenuated glycolysis by inhibiting hexokinase and GAPDH^17^. Furthermore, GAPDH activity was previously reported to be reduced in AD patients^33^. The role of GAPDH in apoptosis has already been described in neurodegenerative disorders^34^. Although GA induced the expression of GAPDH in the present study, we previously reported that GA inactivated GAPDH in a neuronal culture system^8^. ACAC recovered from GA-induced cell death, suggesting that abnormalities in glycolysis may be involved in the neurotoxicity mechanism. GAPDH inactivation due to intracellular GA production further increases intracellular GA concentrations, resulting in increased GA-AGE production and nerve cell toxicity. Thus, GA-AGEs may be one of the general causative agents of the development of neurodegenerative diseases, such as AD.

The brain is in direct contact with the CSF. Biological alterations that reflect pathophysiological processes in the brain are reflected in the CSF. Evidence obtained over the past 20 years has indicated that Aβ42 levels in the CSF of AD patients are significantly lower than those in age-matched healthy elderly controls, whereas total tau and p-tauT181 levels are significantly higher^35^. Furthermore, the levels of other AD biomarkers, such as VEGF^13^ and TGF-β1^36^, were also found to be higher in the CSF of AD patients. Shuvaev *et al*. reported that glycation was greater in the CSF of AD patients than in age-matched controls^37^. In our study, GA decreased Aβ42 levels and increased total tau and p-tauT181 levels in culture media and also increased the intracellular levels of total tau, p-tauT181, VEGF, and TGF-β in SH-SY5Y cells. We were unable to determine why GA decreases Aβ42 levels and increased p-tauT181 levels in culture media. GA may have had effects on the post-translational modifications (e.g., phosphorylation in the early stage) of p-tauT181, but not on the expression and/or secretion of Aβ42 through glycolysis inhibition. Further studies are needed in order to elucidate the mechanisms underlying GA-induced AD-like alterations in diagnostic marker levels. In addition, Glu-AGEs did not induce cytotoxicity, but caused tau-phosphorylation in a culture system^38^. These findings suggest that high levels of glycation and/or GA may be mimicked by AD CSF alterations and accompanied by numerous neuropathological consequences due to GA-AGEs rather than Glu-AGEs.

GA is derived from two distinct pathways, 1) a glycolytic pathway and 2) fructose metabolism pathway^39^. 1) The glycolytic intermediate glyceraldehyde-3-phosphate is generally catabolized by GAPDH. As discussed above, the addition of GA to neuronal cells led to a decrease in GAPDH activity, suggesting a feed-forward mechanism; the GA-AGE-induced inactivation of GAPDH may further stimulate the generation of GA-AGEs. Further studies are needed in order to elucidate the exact mechanisms underlying GAPDH-related neurotoxicity by GA. 2) Under hyperglycemic conditions, an increase in intracellular glucose has been shown to stimulate the polyol pathway to generate fructose in insulin-independent tissues, including the brain and nerve tissue^40^. Fructose is phosphorylated to fructose-1-phosphate and then catabolized to dihydroxyacetone phosphate and GA by liver aldolase B^41^, and GA promotes the formation of GA-AGEs. Moreover, previous studies reported that aldolase B was not expressed in the rat brain^42^, whereas fructose-1-phosphate cleavage (aldolase) activity has been detected in the human brain^43^.

Although the exact mechanisms underlying the target of GA-AGEs and its downstream signaling pathway currently remain unclear, the measurement of GA-AGE levels in the CSF and/or serum may be a useful biomarker for the early detection of AD.

## Materials and Methods

### Materials

SH-SY5Y human neuroblastoma cells were purchased from ECACC (The European Collection of Cell Cultures). Dulbecco’s Modified Eagle Medium (DMEM) and ACAC were obtained from Sigma-Aldrich (St. Louis, MO, USA). GA was from Nacalai Tesque, Inc. (Kyoto, Japan). All other chemicals not indicated were purchased from Wako Pure Chemical Industries, Ltd. (Osaka, Japan).

### Cell culture, cell counting, cell viability, detection of apoptosis, and culture media analyses

Cells were cultured in DMEM on 6-well plates for 24 h, followed by the addition of various concentrations (0, 0.3, 0.7, and 1 mM) of GA, and all assays were performed after a further incubation for 24 h. Cell viability was assessed using Cell counting kit-8 (Dojin, Kumamoto, Japan) according to the manufacturer’s instructions. Apoptosis was examined by fluorescent microscopy using Vybrant® Apoptosis Assay Kit #4 (Invitrogen Corporation, CA, USA), in which early apoptotic cells were detected with green fluorescent YO-PRO®-1. In medium analyses, an aliquot of medium was centrifuged (600 g, 5 min, 4 °C) followed by recentrifugation of the supernatants (21,000 g, 10 min, 4 °C), and the resulting supernatants were used. Aβ42, total tau protein, and p-tauT181 protein were examined by ELISA kits (for the tau protein, its phosphorylated form, and Aβ42, the kit was obtained from Invitrogen Corporation, CA, USA). Lactic acid concentrations were measured by Determiner-LA (Kyowa Medex, Tokyo, Japan). In ACAC experiments, cells were treated with ACAC 15 min before the addition of GA.

### Preparation of an anti-GA-AGE antibody

An immunoaffinity-purified anti-GA-AGE antibody was prepared as described previously^31^. The immunoaffinity-purified anti-GA-AGE antibody did not recognize well-characterized AGE structures, such as CML, *N*^*ε*^-(carboxyethyl)lysine (CEL), pyrraline, pentosidine, argpyrimidine, imidazolone, glyoxal-lysine dimers (GOLD), methylglyoxal-lysine dimers (MOLD), or glyceraldehyde-derived pyridinium (GLAP). Furthermore, it did not recognize AGEs, such as Glu-AGEs and fructose-derived AGEs (Fru-AGEs), the structures of which are unknown^31^,^44^. Instead, the anti-GA-AGE antibody specifically recognized unique unknown GA-AGE structures.

### Slot blotting analysis for GA-AGEs

Cells were harvested and homogenized after being treated for 24 h with GA. An equal amount of protein was applied to a Hybri-SLOT apparatus (Gibco BRL) and transferred to a nitrocellulose membrane (Whatman) by vacuum filtration. After blocking with 3% bovine serum albumin for 1 h at room temperature, samples were incubated with the anti-GA-AGE antibody at 4 °C overnight, followed by incubation with an anti-rabbit IgG antibody (Sigma Aldrich). Antibody-bound protein bands were detected using a BCIP-NBT Kit and densitometrically analyzed.

### Real-time reverse transcription-polymerase chain reaction (real-time RT-PCR)

Total RNA was isolated using ISOGEN (Nippon Gene, Tokyo, Japan) and complementary DNA was synthesized with the Exscript RT reagent kit (Takara Bio Inc., Shiga, Japan). Real-time RT-PCR was performed using the Smart Cycler II system (Cepheid, CA, USA) and SYBR Premix Ex Taq reagent (Takara Bio Inc., Shiga, Japan). β-Actin was used as an internal control. The following primers were designed to produce mRNA-specific amplification products: β-actin, 5′- TCC ACC TTC CAG CAG ATG TGG -3′, and 5′- GCA TTT GCG GTG GAC GAT -3′; GAPDH, 5′- TGG GCT ACA CTG AGC ACC AG-3′, and 5′- CAG CGT CAA AGG TGG AGG AG-3′ ; VEGF, 5′- TGC AGA TTA TGC GGA TCA AAC C-3′, and 5′- TGC ATT CAC ATT TGT TGT GCT GTA C-3′; TGF-β, 5′- GCG TGC TAA TGG TGG AAA CC-3′, and 5′- CGG AGC TCT GAT GTG TTG AAG A-3′.

### Measurements of intracellular tau and phosphorylated tau

Intracellular concentrations of tau and phosphorylated tau proteins were measured as follows: Cells were dissolved in extraction buffer containing 10 mM Tris HCl (pH 7.4), 100 mM NaCl, 1 mM EDTA, 1 mM EGTA, 1 mM NaF, 20 mM Na_4_P_2_O_7_, 2 mM Na_3_VO_4_, 1% Triton X-100, 10% glycerol, 0.1% SDS, 0.5% deoxycholate, and complete (Roche Diagnostics, Basel, Switzerland), a protease inhibitor, followed by incubation for 30 min on ice. After being centrifuged (15,000 g, 10 min, 4 °C), supernatants were applied to the ELISA kit. Intracellular protein concentrations were measured using Bradford ULTRA (Expedeon, Cambridgeshire, UK).

### Statistical analysis

Data are expressed as the mean ± SD, and were examined by a one-way analysis of variance (n = 3 or n = 6). More than two experiments were performed and similar results were obtained. P values less than 0.05 were considered to be significant.

## Additional Information

**How to cite this article**: Koriyama, Y. *et al*. Glyceraldehyde caused Alzheimer’s disease-like alterations in diagnostic marker levels in SH-SY5Y human neuroblastoma cells. *Sci. Rep*. **5**, 13313; doi: 10.1038/srep13313 (2015).
